# Syntheses of Prussian Blue Pigment Following 18th-Century Methodologies: Factors Influencing Product Purity and Syntheses Yields

**DOI:** 10.1021/acsomega.4c11328

**Published:** 2025-03-13

**Authors:** Mônica Grôppo Parma, Heloísa Beraldo, Isolda de Castro Mendes

**Affiliations:** † Departamento de Química, Instituto de Ciência Exatas, Universidade Federal de Minas Gerais, Av. Antônio Carlos, 6627, 31270-901 Belo Horizonte, Minas Gerais, Brazil

## Abstract

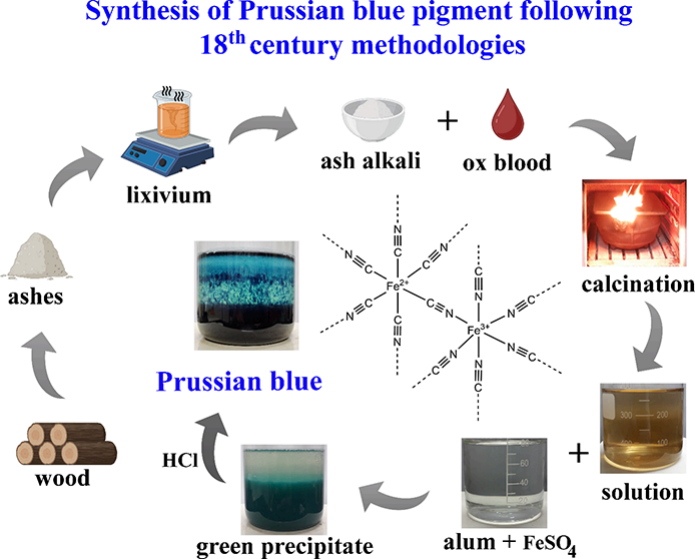

In the present work, Prussian blue syntheses were performed based on the methodologies described in “The Handmaid to the Arts Teaching”, v.1 and 2 (1758). Since calcination is considered the crucial step in the synthesis, the reagents and parameters of this step, such as time, temperature, and calcination vessel, were varied. Attempting to reproduce the calcination as closely as possible to the original description, in addition to commercial reagents (potassium carbonate, blood meal, alum, and iron(II) sulfate), reagents such as *in natura* ox blood and ash alkali were employed. The ox blood was dried in a muffle furnace and then macerated. The ash alkali was obtained by Candeia wood (*Eremanthus erythropappus*) ash lye, and its composition was characterized by the compounds K_2_CO_3_, KHCO_3_, K_2_SO_4_, KCl, K_2_O, and SiO_2_. In the calcination step, alumina or iron vessels were used. It was observed that the iron vessel enhanced the reaction yield, as did the amount of HCl added in the last step of the synthesis. The results of the calcination time and temperature parameters are related to the material of the vessel used; however, a minimum temperature of 400 °C was necessary for the formation of Prussian blue. Impurities of amorphous carbon, KCl, alum, sulfates/sulfides, and aluminum hydroxide were also identified.

## 1 Introduction

Prussian blue, Fe_4_[Fe(CN)_6_]_3_, was the first coordination compound to be synthesized in the early 18th-century, almost two centuries before this class of compounds began to be studied by Alfred Werner (1866–1919). This event was of great relevance to the artistic world since this pigment has high hiding power and was cheaper when compared to ultramarine blue (Na_8–10_Al_6_Si_6_O_24_S_2–4_), the pigment most highly regarded by artists at the time.^1^ Today, Prussian blue and its analogues continue to be a source of great interest due to their properties that generate applications in the medical as therapy and diagnostic imaging,^2,3^ biosensors,^4^ catalytic activities applied in environmental and energy,^5,6^ electrochemical,^7−9^ analytical and laboratory processes^10^ and artistic areas,^11^ among others.^12−14^

Two forms of Prussian blue are reported in the literature. The soluble form is represented by the formula AFe[Fe(CN)_6_]•yH_2_O, with y = 0–5; A = K^+^, Na^+^ or NH_4_^+^, and contains alkali metal ions or small molecules in its structure. The insoluble form is represented as Fe_4_[Fe(CN)_6_]_3_•xH_2_O, with x = 5–18.^15^ The terms “soluble” and “insoluble” are used only to highlight the presence or absence of alkali ions in the structure of the complex. Regarding solubility in water, both species are insoluble and have a colloidal nature.^16,17^

The story behind the discovery of this pigment is still not completely known. However, historical sources^18−21^ indicate that the first synthesis of Prussian blue occurred accidentally when the paint manufacturer Johann J. Diesbach (1670–1748) prepared a purple lacquer based on cochineal, alum, Mars vitriol, and potash, called Florentine lacquer. The potash used was a purified residue from the horn oil of Johann C. Dippel (1673–1734), a well-known alchemist of the time. While carrying out his procedures, Diesbach observed the appearance of a blue color, indicating the formation of an unexpected compound called *Berlin blau*, or Prussian blue.^20^

**Figure 1:**
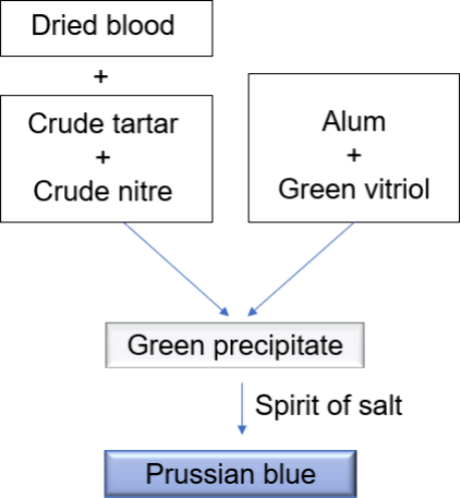
Flowchart of the first Prussian blue synthesis.

The first publication with the methodology for synthesizing Prussian blue was sent “anonymously” to the journal Philosophical Transactions of the Royal Society of London, and published by John Woodward (1665–1728) in 1724.^22^ The new pigment synthesis was based on five main reagents: *crude tartar*, *crude nitre*, dried ox blood, green vitriol, and alum (Figure 1). Initially, calcination should be performed with a mixture of *crude tartar* and *crude nitre*. The resulting product should then be calcined again with dried ox blood, a stage considered the most important in the synthesis.

Due to the lack of systematization in the nomenclature of materials during the earliest publications on the synthesis of Prussian blue, providing a definite interpretation of the chemical composition and purity of the compounds is challenging.^23^ For instance, “*alum*” may refer to potassium–aluminum double sulfate or ammonium-aluminum double sulfate.^24^ Green vitriol, synonymous with Mars vitriol, corresponds to iron(II) sulfate. The term “*nitre*” refers to potassium nitrate; “*tartar*” to potassium hydrogen tartrate; “*sal tartari*” to potassium carbonate; and “*spirit of salt*” to hydrochloric acid.^24^ Information on preparation techniques and properties described in treatises and dictionaries of the time, such as color, grain size (grinding), and density (decantation), may assist in accurately meaning each term. Different nomenclatures were also applied to compounds with varying degrees of purity.^24^

John Brown (18th-century, 1735), a scientist and then reviewer of the Philosophical Transactions, verified the veracity and reproducibility of the unprecedented synthesis of Prussian blue.^25^ In addition to testing the method, Brown performed several experiments replacing green vitriol with other metal salts such as Ag, Hg, Cu, Bi, and Pb, to prove that iron was essential for obtaining the blue pigment. This is currently considered the first research on compounds analogous to Prussian blue.^19,25^

The stoichiometry of the Prussian Blue synthesis, according to 18th-century methodologies, is not trivial. The limiting reagent of the reaction is the product formed in the calcination step. The calcination parameters and the materials used in this stage are essential for a successful synthesis. Other studies reproducing the first syntheses of this pigment using commercial reagents and porcelain vessels in the calcination stage have already been reported.^26−28^ Only two historical references present yields,^25,29^ and this information is not mentioned in modern papers. To our knowledge, the impact of temperature, time, and vessel material used during calcination on the products and their yields has not been studied so far.

Based on research into the methodologies for synthesizing Prussian blue over a hundred years since its first publication, two procedures from 1758 were chosen for practical study.^29^ The synthesis was reproduced to emphasize the challenges in obtaining a commercial yield of Prussian blue, its potential byproducts, as well as the influence of the reagents and the importance of the calcination step. This is the first work to report on the synthesis of Prussian blue in a way more faithful to what was done in the 18th century. Examining the impurities associated with the Prussian blue such as sulfur and aluminum could be clues that can differentiate a pigment produced by current methods from one in the 18th century, as well as the particle size. This is especially important for the conservation and dating of cultural heritage.

## 2 Materials and Methods

The reagents AlK(SO_4_)_2_·12H_2_O (Synth), FeSO_4_·7H_2_O (Sigma-Aldrich) and HCl (CRQ) used in the syntheses were obtained commercially with a high degree of purity since they are not present in the calcination step between the animal matter and the alkali. The animal matter used was *in natura* ox blood acquired in a slaughterhouse. The blood meal (*Cevas Iscas*) was used without further treatment as purchased from a commercial location. The ox blood was dried in porcelain crucibles (H: 58 mm, D: 50 mm, d: 30 mm) in a muffle furnace at T = 150 °C for 2 h and subsequently macerated in an agate mortar. The alkali was obtained from Candeia wood (*Eremanthus erythropappus*) ash lye according to the methodology of Dossie (1766) published by Velloso (1798, p. 65–67),^30^ and K_2_CO_3_ (Carlos Erba).

The products were characterized using Raman light spectroscopy, infrared spectroscopy (IR), energy-dispersive X-ray fluorescence spectroscopy (EDXRF), scanning electron microscopy (SEM-EDS), X-ray diffraction (XRD) and thermogravimetric analysis (TGA). The conditions used for each analysis are described in the Supporting Information.

### 2.1 Ash Alkali

**Figure 2:**
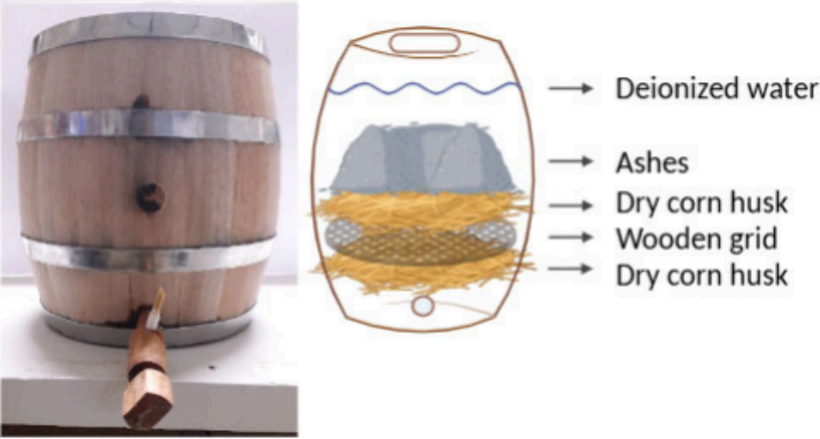
Assembly diagram of the ash-leaching wood container.

One kg of Candeia wood ash was placed inside a previously prepared wooden barrel (Figure 2) and deionized water (1.3 L) was added until filling 3/4 of the container’s volume. The mixture was left to stand for 20 h and then the first lixivium was removed. Another 500 mL of deionized water was added, and the ashes were stirred. The mixture was left to stand again and after 1 h the second lixivium was removed. The last procedure was repeated once more. In total, three lixiviums were collected, providing a total volume of 1.65 L (orange solution, pH 12), and evaporated by heating in an iron vessel. The obtained residue, still moist, was transferred to porcelain crucibles and dried in a muffle furnace at 150 °C. After drying, the ash alkali residue was macerated, presenting a strong odor and light brown color. Hygroscopic product. Yield: ∼ 56 g.

### 2.2 Prussian Blue

Historical documentary research was conducted in manuals, books of secrets, dictionaries, and treatises from the 18th and 19th centuries that describe the synthesis of Prussian blue pigment. To better understand how the synthesis conditions (temperature, time, vessel, reagents, and their proportions) could influence the yield and products formed, syntheses were performed varying these parameters (Table 1). The syntheses were based on the English treatise The Handmaid to the Arts Teaching, v.1 and 2 (1758), written by Robert Dossie, and were performed using 30 g of dried blood. A transcription of the recipes can be found in the Supporting Information. The reagents used by Dossie are dry ox blood, pearl-ashes, green vitriol, alum, and spirits of salt in the mass proportions of 3:1:1:2:2 and 8:8:1:16:3. In the calcination stage, the use of covered metal crucibles or ceramic crucibles (earthen-pots) was specified.

**Table 1:** Syntheses of Prussian Blue based on Dossie, 1758, Mass Ratios Blood:Alkali 3:1 and 1:1 (I6)

Synthesis	Time (min)	Calcination temp. (°C)^a^	Yield (mg)	Recipient	Reagents	Final separation method
**I1**	75	to 350	0	Iron	Blood meal + K_2_CO_3_	Centrifugation
**I2**	60	to 400	340	Iron	Blood meal + K_2_CO_3_	Centrifugation
**I3**	90	to 400	210	Iron	Blood meal + K_2_CO_3_	Centrifugation
**I4**	60	to 400	150	Iron	Blood meal + K_2_CO_3_	Centrifugation
	30	to 450				
**I5**	60	to 400	166	Iron	Blood meal + K_2_CO_3_	Filtration
	30	to 450				
**I6**	60	to 400	13.856	Iron	Blood meal + K_2_CO_3_	Filtration
	30	to 450				
**I7**	60	to 400	290	Iron	Dried ox blood + ash alkali	Centrifugation
	30	to 450				
**I8**	60	to 400	255	Iron	Dried ox blood + K_2_CO_3_	Centrifugation
	30	to 450				
**I9**	60	to 400	265	Iron	Dried ox blood + ash alkali	Centrifugation
	30	to 450				
**I10**	60	to 400	2	Iron	Blood meal + ash alkali	Centrifugation
	30	to 600				
**I11**	60	to 400 to 600 to 700	1	Iron	Blood meal + ash alkali	Centrifugation
	40					
	20					
**A1**	60	to 400	887	Alumina	Dried ox blood + ash alkali	Centrifugation
	30	to 450				
**A2**	30	to 400	5	Alumina	Dried ox blood + K_2_CO_3_	Decantation
	150	to 450				
**A3**	90	to 400	40	Alumina	Blood meal + ash alkali	Decantation
	105	to 600				
**A4**	150	to 540	<1	Alumina	Dried ox blood + K_2_CO_3_	Decantation
	120	to 600				

a Initial temperature 25 °C.

In all the syntheses conducted, the animal matter and alkali were macerated together and placed in covered vessels (either alumina or iron) for calcination in a muffle furnace, under an exhaust hood. The alumina crucible (H: 68 mm, D: 58 mm, d: 58 mm) was covered with an alumina lid and the iron vessel (H: 73 mm, D: 100 mm, d: 45 mm) with an iron lid. There was an increase in the volume of the calcined material, emission of gases, a strong odor, and flame formation. At the end of the calcination time, the material was solubilized in 300 mL of boiling deionized water. The mixture was boiled with sporadic stirring for 45 min and then filtered (pH 12–13). In the syntheses in which alumina vessels were used, the calcined material was transferred to a beaker with boiling deionized water, while in the syntheses carried out in the iron vessel, the solubilization, and boiling step was performed directly in the iron vessel.

In parallel with the filtration, a solution of alum + iron(II) sulfate (pH 3) was prepared and slowly added to the calcination filtrate (for more details, see the Supporting Information). There was effervescence and the immediate formation of a white precipitate, which rapidly turned green. The precipitate decanted and the supernatant oxidized quickly, becoming cloudy and changing to orange, evidencing iron(III) hydroxide and oxide formation. The pH varied during this process from basic to close to neutral. Therefore, we can infer that aluminum oxides and hydroxides can also be formed.^31,32^ The mixture was left to stand, and all precipitates were 3-fold washed after decantation. The last removed supernatant appeared clear (pH 4–5), and the precipitate was blue-greenish.

After the third wash, 37% HCl was added to the precipitate. The mixture was again left to stand and all products obtained were 3-fold washed after decantation. In the syntheses using the iron vessel, decantation of the precipitate was rapid, whereas in the syntheses carried out in the alumina vessel, the process was slower. This suggests the formation of grains with different average sizes. Tests for product separation (filtration, centrifugation, or decantation) were evaluated (Table 1). After separation, the products were dried in desiccators over silica gel.

## 3 Results and Discussion

### 3.1 Historical Documentary Analysis

Documentary research reveals that, until the early 19th century, numerous syntheses and studies of Prussian blue were described, utilizing various animal materials, different methods for obtaining alkali, and varying reagent proportions. These factors can affect the purity and yield of the products.

Table S1 (Supporting Information) reports the work of Kirby and Saunders^27^ together with other references reporting the synthesis of Prussian blue from its first publication in 1724 to 1837 when the first reference citing the use of coke, potash, and metallic iron as reagents was found. These inorganic reagents were proposed to synthesize potassium ferrocyanide from the preparation of cyanogen, (CN)_2_, described in 1814 by Joseph Gay-Lussac (1778–1850),^33^ which characterizes the modern syntheses of Prussian blue. An overview of the recipes found in sources from 1845 to the beginning of the 20th century can be found in Reus et al.^28^

In the first published synthesis (1724), Woodward specifies that the alkali used was formed from the calcination of *crude nitre* (KNO_3_) and *crude tartar* (potassium bitartrate).^22^ Another method for obtaining this compound involved the leaching of vegetable ashes, as described in a synthesis of 1758.^29,30^ The book Alografia dos alkalis fixos vegetal ou potassa (1798), by Velloso,^30^ contains a compilation of procedures for the production of alkalis in Europe and America during the 18th-century, and is a good example of the importance and diversity of plant sources used for alkalis production. The leaching of ashes from certain plants was intended to yield potassium carbonate, so plant species with a higher potassium content would be ideal.

As for animal matter, in addition to the ox blood mentioned by Woodward, scientific experiments on the synthesis of various animal matters were carried out to discuss the formation and origin of the Prussian blue color. In the first study, a review of Woodward’s article, Brown (1724) reports that other animal parts such as horns and hooves could also be used.^25^ The use of these different animal materials was only described again in chemistry or science treatises from 1788 onward.^34−37^ Only syntheses using ox blood as animal matter were found in the art treatises and books of secrets analyzed. Brown^25^ also describes the production of a blue pigment with a light hue when alum is not used, but does not rule out its use in the synthesis discussion. The use of alum in the first synthesis of Prussian blue is linked to its ability to precipitate organic dyes in the form of lakes, since Prussian blue was obtained unexpectedly during an attempt to produce an organic lake.

Table 2 presents the reagent’s mass proportions and the synthesis yields of Prussian blue cited by Brown and Dossie. When studying the synthesis published by Woodward, Brown used “salt of tartar” (potassium carbonate) as the alkali instead of the product obtained by calcination between *crude tartar* and *crude nitre*. The synthesis yield was 25% concerning the mass alkali. Dossie (v.2), in turn, claims to describe the original recipe published by Woodward, but presents a smaller proportion of green vitriol and acid (spirit of salt), and uses pearl-ashes as the alkali. The synthesis yield obtained by Dossie was ∼19% concerning the alkali mass.

**Table 2:** Mass Proportion of Reagents for the Synthesis of Prussian Blue

Reference	Dried ox blood	Alkali	Green vitriol	Alum	Spirit of salt^a^	Yield
Woodward, 1724	4	4 *crude tartar*, 4 *crude nitre*	1	8	2–3	-
Brown, 1724	4	4 *sal tartari*	1	8	2–3	1
Dossie, 1758, v.1	12	4 pearl-ashes	4	8	8	-
Dossie, 1758, v.2	4	4 pearl-ashes	0.5	8	1.5	0.75

a HCl 37% solution mass.

One challenge in interpreting these 18th-century syntheses is the lack of information regarding key parameters such as temperature and reaction time. At that time, synthesis steps were carried out in brick kilns built for this purpose, as specified by Dossie.^29^ Although these treatises provide instructions on constructing the kilns, they do not mention the temperature achieved. In the case of Prussian blue, the step carried out in these kilns is calcination. None of the works specify the calcination temperature and Brown is one of the few authors who reports the calcination time, and claims to have done it in 2 h.^25^ Other authors emphasize that ceramic or metallic vessels (iron, copper, or tin, called pewter boilers) should be used in this step.^29,36,38,39^

### 3.2 Synthesis and Characterization

#### 3.2.1 Ash Alkali

Alkali is an important point for the Prussian blue synthesis based on 18th-century methodologies, since depending on its method of obtaining, different impurities may be associated with it. Using semiquantitative elementary techniques, the Candeia wood ash before the leaching process presented ∼25% K in its composition, while the product obtained presented ∼86% K, evidencing the efficiency of the leaching process. In addition to potassium, other elements qualitatively identified by EDS in the ash alkali were C, O, Na, Al, S, and Cl, and traces of Mg and Si.

**Figure 3:**
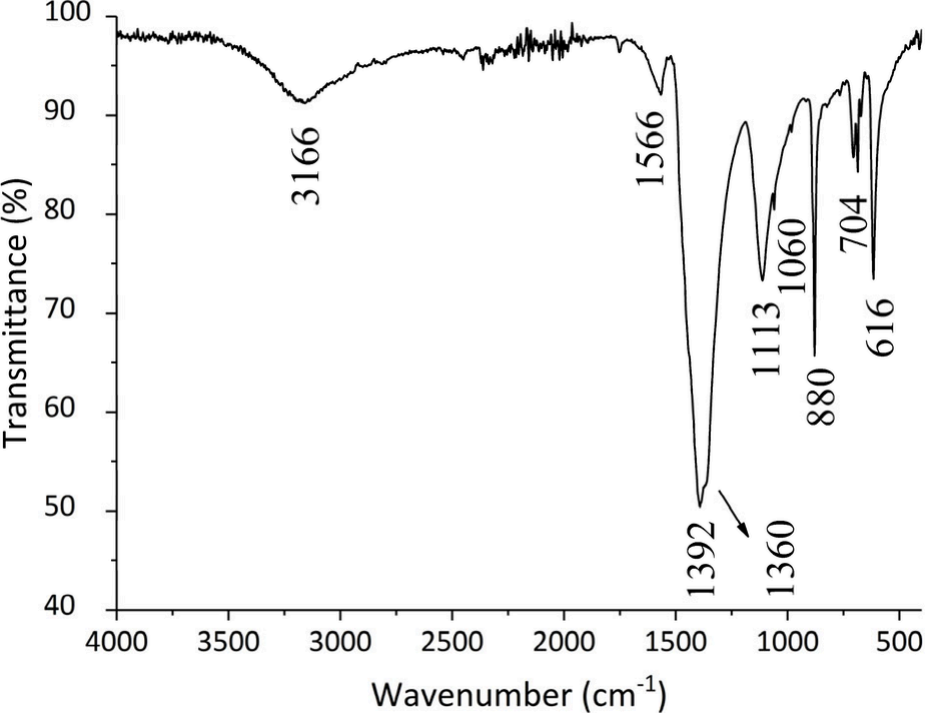
Infrared spectrum (ATR) of ash alkali.

In the infrared spectrum (Figure 3), bands characteristic of the compound K_2_CO_3_ were observed at 3166 (br) cm^–1^, 1360(sh) cm^–1^, 1060(sh) cm^–1^, 880(s) cm^–1^, and 704(w) cm^–1^.^40^ The overlapped bands at 1392(vs) cm^–1^ and 1360(sh) cm^–1^ are attributed to the asymmetric stretching bands of the carbonate. Bands at 1113(m) cm^–1^ and 616(m) cm^–1^ correspond to sodium or potassium sulfate.

By the XRD technique (Figure S1, Supporting Information), the compounds K_2_SO_4_, KCl, K_2_O, KOH and KHCO_3_ (peaks at 2θ = 12.27°; 12.82°; 23.00°; 32.13°; 32.29°; 32.61°; 35.17°; 38.60°; 39.02°; 41.30°; 41.61°; and 54.99°) present in the ash alkali were identified. K_2_CO_3_ was not identified by XRD, which indicates its amorphous form.

#### 3.2.2 Prussian Blue

Fifteen syntheses of Prussian blue pigment were performed according to the methodology described by Dossie (v.1, 1758), varying the reagent parameters, calcination time, maximum calcination temperature, vessel used in this step and final product separation method (Table 1). After analyzing the results, selected parameters were chosen to perform a synthesis of Prussian blue (synthesis I6) according to the methodology described by Dossie (v.2, 1758). The mass proportions of the reagents used in the two methodologies are described in Table 2.

Among the syntheses performed, three did not produce the desired result. In synthesis I1 (*T*_max_ = 350 °C, iron vessel), no flame was observed during the calcination step, and the mixing of the calcination filtrate and ferrous sulfate/alum solutions did not result in any solid product. In two syntheses, I2 (*T*_max_ = 400 °C, iron vessel) and A1 (*T*_max_ = 450 °C, alumina vessel), the mixing step of the solutions generated a large amount of foam inside the beaker, and the precipitate formed had a dark green color with a foul odor, different from the other syntheses. After washing, decanting, and adding HCl the precipitate color does not change.

The other syntheses indicated that the maximum calcination time and temperature are related to the vessel used in this step. For example, a calcination time of 90 min up to 450 °C was sufficient to form Prussian blue using an iron vessel. Under these same conditions, using an alumina vessel (synthesis A1), the reaction did not achieve the desired product, whereas, with the calcination time of 180 min up to 450 °C, the Prussian blue pigment was obtained.

This fact may be related to the thermal conductivity of the two materials. Iron thermal conductivity (80 W m^–1^ K^–1^) is approximately twice that of alumina (39 W m^–1^ K^–1^). The lower the thermal conductivity value, the lower the heat transfer between the vessel and the material to be calcined,^41,42^ which suggests that more time is needed for the reaction to occur. The greater thermal stability of alumina vessels does not appear to have influenced the synthesis.

Regarding the yield of synthesis, starting from 30 g of animal matter, few milligrams were obtained for the syntheses with a 3:1:1:2:2 ratio of blood:alkali:vitriol:alum:acid, a lower yield was obtained compared with those reported by Brown and Dossie (Table 2). On the other hand, the synthesis performed with the 1:1:1/8:2:3/8 ratio (synthesis I6) generated a mass of approximately 14 g of product, with a higher yield of 46%.

In synthesis I6 a lower ratio of alum:HCl (8:1.5) was used. The acid the last stage of the synthesis has the function of dissolving impurities with sulfides, iron, and aluminum hydroxides that have formed together as products,^43^ so that the addition of a significantly smaller amount of acid can favor the presence of these impurities, which seems to have happened and led to the increase in the yield of synthesis I6 and its discrepancy with the others.

For the product and impurities characterization obtained in the syntheses, elemental analysis, spectroscopic, and thermogravimetric techniques were used. The EDS, XRD, IR, Raman, and TGA results for each sample are presented in Table 3.

**Table 3:** EDS, XRD, IR, Raman, and TGA Characterization of the Synthesis Products

Synthesis	EDS	XRD^a^	IR	Raman	Residue TGA (%)
**A1**	C, N, O, S, Fe, Al, Cl, Na, Mg, Si	PB	Fe-CN, water, sulfate/sulfide	Fe-CN, carbon	19.50
**A2**	C, N, O, K, S, Fe, Al, Si, Na, Cl	PB	Fe-CN, water	Fe-CN, carbon	-
**A3**	C, N, O, K, S, Fe, Al, Si, Cl, Na, P	PB,	Fe-CN, water, sulfate/sulfide	Fe-CN, carbon	-
		KAl(SO_4_)_2_			
**A4**	C, N, O, K, S, Fe, Al, Cl, Na, P, Si	PB, KCl,	Fe-CN, water, sulfate/sulfide	Fe-CN, carbon	-
		KAl(SO_4_)_2_			
**I1**	-	-	-	-	-
**I2**	C, N, O, S, Fe, Cl, K, Al, Si	PB	Fe-CN, water, sulfate/sulfide	Fe-CN, carbon	20.40
**I3**	C, N, O, K, S, Fe, Al, P, Cl, Si	PB	Fe-CN, water	Fe-CN, carbon	45.85
**I4**	C, N, O, K, S, P, Fe, Si, Al, Cl	PB	Fe-CN, water	Fe-CN, carbon	51.09
**I5**	C, N, O, K, S, Fe, Al, Si, Cl, P	PB	Fe-CN, water, sulfate/sulfide	Fe-CN, carbon	48.77
**I6**	C, O, S, Al, N, P, K, Si, Fe	PB	Fe-CN, water, sulfate/sulfide	Fe-CN, carbon	42.92
**I7**	C, N, O, K, S, Fe, Al, Si, Cl, P	PB	Fe-CN, water	Fe-CN, carbon	51.66
**I8**	C, N, O, K, S, Fe, P, Al, Cl, Si	PB	Fe-CN, water	Fe-CN, carbon	47.77
**I9**	C, N, O, K, S, Fe, Al, Si, Cl, Na, P	PB	Fe-CN, water	Fe-CN, carbon	50.15
**I10**	C, N, O, S, Fe, Al, Si, Na, Mg, P, K, Cl	PB	-	Fe-CN, carbon	-
**I11**	C, N, O, S, Fe, Al, Si, Na, Mg, P, K, Cl	PB	-	Fe-CN, carbon	-

a PB: Prussian blue.

In general, the presence of Na, P, S, Al, Cl, and Si was observed in practically all synthesized products, indicating the presence of impurities. Compounds with K, such as alum and KCl, and amorphous carbon were also confirmed as impurities.

Regarding synthesis I6 (8:1.5 alum:HCl ratio), elemental analysis suggested the presence of Al, S, K, and Cl in a greater proportion than iron. For this reason, the final washing step (3x decantation/washing) was repeated twice. The elemental analysis results of the product from the first to the second wash did not show K and Cl, probably due to the high solubility of the KCl compound in water (33 g/100 mL). Sulfur and aluminum remained present, indicating that these elements constitute insoluble compounds such as sulfides, oxides, or hydroxides. In the third wash, the quantities of Fe, Al, and S remained almost unchanged, and the product was not once more washed.

Through XRD analysis, it was possible to identify Prussian blue in all analyzed samples (Figures S2 and S3). This complex crystallizes in space group 225, with face-centered cubic symmetry *Fm*3̅*m*. Additional reflections at *d* = 5.869 (111) and *d* = 3.065 (311) are reported in the literature in the diffractogram of the soluble form of Prussian blue, indicating a slightly different crystal structure from the insoluble form.^44^ The presence of these two reflections, confirming the formation of the two forms of Prussian blue was not possible, perhaps due to the low relative intensities of the peaks of the soluble form and the presence of fluorescence.

**Figure 4:**
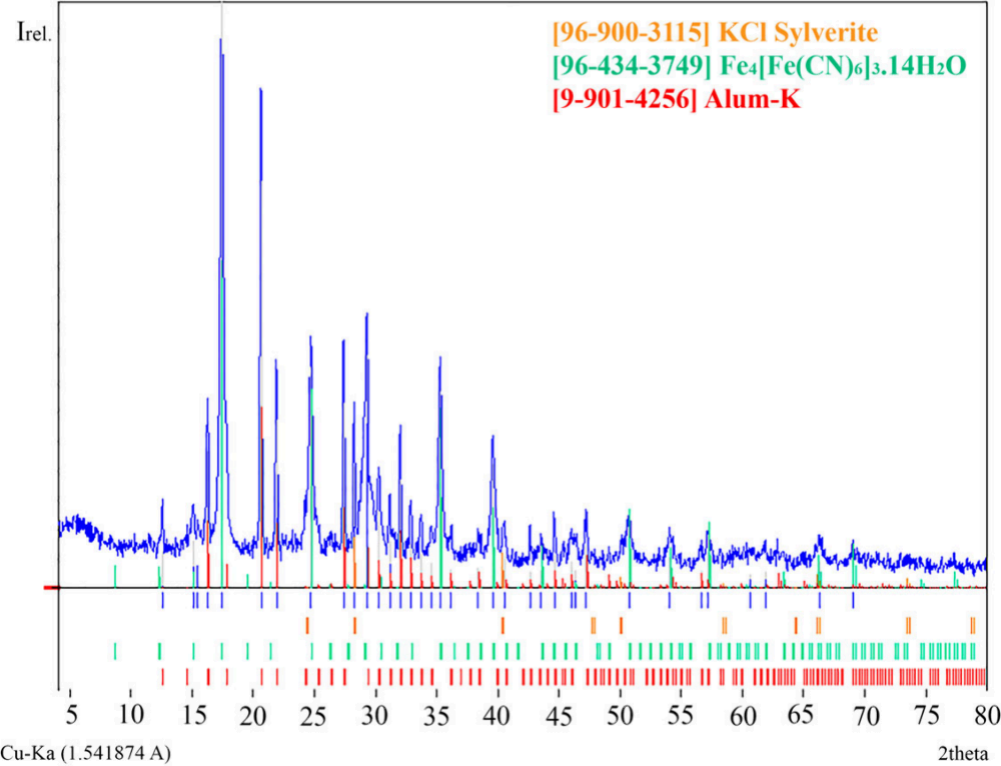
Diffraction pattern of sample A4.

**Figure 5:**
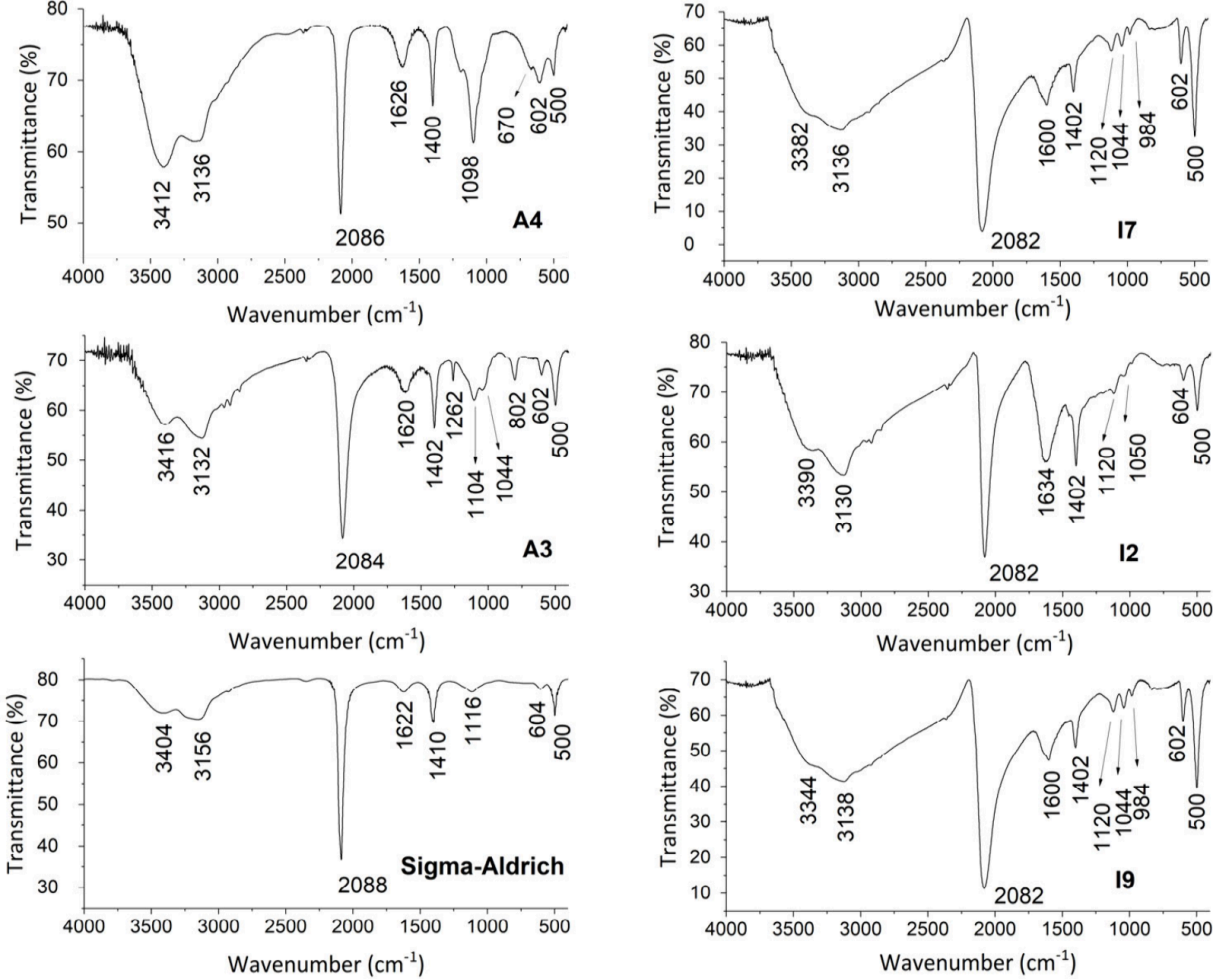
Baseline-corrected infrared spectra (KBr pellets) of samples A4, A3, I7, I2, I9, and Sigma-Aldrich Prussian blue.

Impurities such as alum and KCl were found in samples A3 and A4 (Figure 4 and S3), while in the other samples, the crystalline phases of the impurities should be below the detection level of the technique (1%) or in amorphous form. In the infrared spectra of most samples, the presence of characteristic bands of sulfate/sulfide groups was observed in the range 1200–800 cm^–1^, with varying intensities (Figure 5).

Comparing the diffractograms of the products (Figures S2 and S3), samples A1, I2 (dark green products), and I6 presented high fluorescence, which may be due to the presence of amorphous compounds formed by the different conditions used in the syntheses. In A1 and I2 synthesis, it could be due to the maximum temperature achieved in the calcination step (*T*_max_= 400 °C), which might have modified the combustion products. In the I6 synthesis, the low proportion of acid/alum used in the last steps might have favored the presence of amorphous compounds formed during the synthesis. Impurities of alumina hydrate (Al_10_O_14_(OH)_2_) and ferrihydrite (Fe_10_O_14_(OH)_2_) together with Prussian blue synthesized by the methodology described by Dossie (1758) were identified as in other works.^26,27^

XRD and infrared spectroscopy observed variations in the particle size of the products depending on the vessel used during the synthesis. The width of the peaks observed in the diffractograms (Figures S2 and S3) of the products synthesized in an iron vessel is larger than that observed in an alumina vessel. In the infrared spectra (Figures 5 and S4), a wider width of the band corresponding to the ν(CN) symmetric stretching at ∼2088 cm^–1^ is also observed for the products synthesized from the iron vessel. The width of the infrared bands is normally associated with the purity of the product and the particle size; the smaller the particle size, the smaller the width of the band.^45^ These observations corroborate what was observed during the syntheses, in which the products synthesized from the iron vessel presented faster decantation.

The images obtained by SEM (Figure S5) did not reveal any differentiation in grain size between the products obtained from different vessels. A heterogeneous morphology of small fragments and compacted blocks was observed in all synthesized products, indicating the formation of small particles.

The surface adsorption of significant amounts of ions, such as aluminum and potassium, as well as the zeolite binding of water used to wash the precipitates may be related to the processes used in the preparation of Prussian blue and to the fact that the product was obtained as a very thin precipitate.^16^ The differences observed in the 3500–3000 cm^–1^ region (Figure 5) suggest different degrees of hydration of the synthesized products. The band attributed to the ν(CN) stretching varied from 2 to 6 cm^–1^ to lower wavenumbers in the spectra of the synthesized products compared to the Sigma-Aldrich standard (2088 cm^–1^). Only I6 showed a shift of 6 cm^–1^ to higher wavenumbers. Although the TGA also indicated a higher degree of hydration of the synthesis products when compared to the standard, the ν(CN) stretching shifts observed in the infrared could not be associated with the different degrees of hydration obtained.

**Figure 6:**
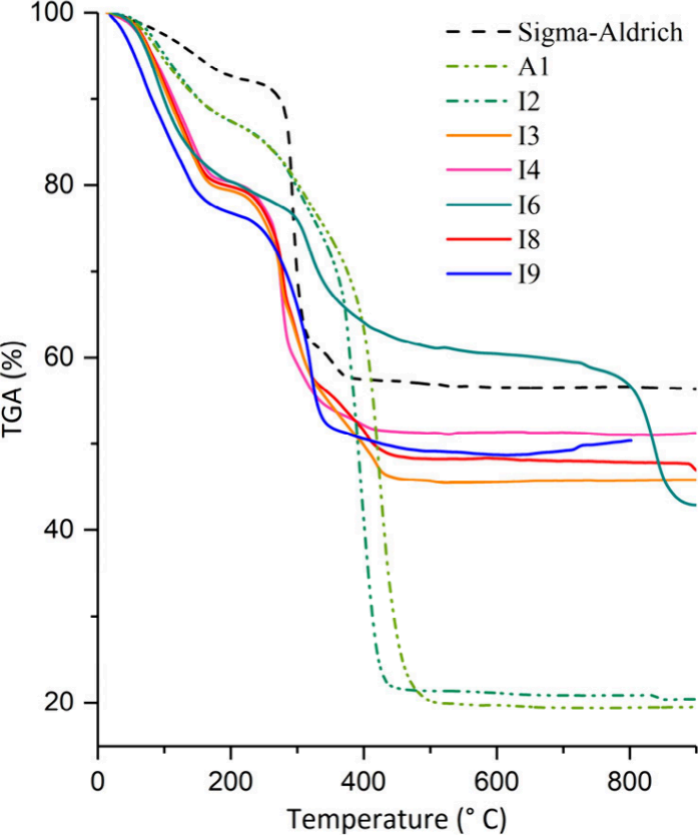
Thermogravimetric analysis curves of Prussian blue pigment samples.

Figure 6 shows the TGA curves of seven samples analyzed with yields higher than 150 mg and one reference sample (Sigma-Aldrich). Four different thermal decomposition patterns can be observed. The first is the Prussian blue sample from Sigma-Aldrich (line **- - -**), which presented a lower percentage by mass in the first loss and a higher percentage of residue than the analyzed synthesis products. The second group of samples refers to syntheses I3, I4, I6, I8, and I9 (lines **–––**), which presented a first mass loss of approximately 20% and final residue between 46% and 51%. The third thermal decomposition pattern corresponds to samples I2 and A1 (lines - -). Both presented products with green color. The final residue mass percentages of these samples were approximately 30% lower than the others, indicating that the proportion of Prussian blue formed was lower and that the combustion of the organic matter was probably not complete due to the maximum temperature during the calcination stage. Among all the samples, I6 was the only one that presented a third mass loss after 520 °C, defining the fourth decomposition pattern.

The first mass loss considered up to 200 °C observed in the TGA curves probably corresponds to water molecules. The second mass loss (200–520 °C) is characterized by an exothermic process related to the oxidation of Fe^II^ ions and the loss of water and cyanide ions from the Prussian blue structure, releasing gases such as (CN)_2_, HCN, NO_*x*_, CO_2_, and CO.^46−48^Table 4 presents the TGA results and theoretical values for different degrees of hydration of Prussian blue. These were used to suggest the degree of hydration and the molecular formula of the synthesized products. The calculations were based on the percentage of the first mass loss since the presence of the impurities suggested in Table 2 makes the calculations based on the mass percentage of the residues impossible.

**Table 4:** TGA Results and Suggested Formulas for SA (Sigma-Aldrich), A1, I2, I3, I4, I6, I7, I8, and I9 and Theoretical Percentages of Mass Losses and Residues of Prussian Blue

Synthesis	1^a^ loss (% m)	2^a^ loss (% m)	3^a^ loss (% m)	Final residue (% m)	Suggested formulas
**SA**	7.38	36.21	-	56.41	Fe_4_[Fe(CN)_6_]_3_·3.8H_2_O
**A1**	12.90	67.46	-	19.50	Fe_4_[Fe(CN)_6_]_3_·7.1H_2_O
**I2**	12.60	66.04	-	20.40	Fe_4_[Fe(CN)_6_]_3_·6.9H_2_O
**I3**	19.66	34.51	-	45.83	Fe_4_[Fe(CN)_6_]_3_·11.7H_2_O
**I4**	19.64	29.12	-	51.24	Fe_4_[Fe(CN)_6_]_3_·11.7H_2_O
**I6**	19.61	19.25	18.22	42.92	Fe_4_[Fe(CN)_6_]_3_·11.7H_2_O
**I8**	19.64	33.43	-	46.93	Fe_4_[Fe(CN)_6_]_3_·11.7H_2_O
**I9**	23,22	26.41	-	50.37	Fe_4_[Fe(CN)_6_]_3_·14.5H_2_O

	% m H_2_O (1^a^ loss)	% m CN (2^a^ loss)	-	% m Fe_2_O_3_ (residue)	Formula
**Theoretical**	0	54.50	-	65.05	Fe_4_[Fe(CN)_6_]_3_
**Theoretical**	7.74	50.29	-	60.02	Fe_4_[Fe(CN)_6_]_3_·4H_2_O
**Theoretical**	12.80	47.53	-	56.73	Fe_4_[Fe(CN)_6_]_3_·7H_2_O
**Theoretical**	20.10	43.55	-	51.98	Fe_4_[Fe(CN)_6_]_3_·12H_2_O
**Theoretical**	23.93	41.46	-	49.49	Fe_4_[Fe(CN)_6_]_3_·15H_2_O

In the Prussian blue structure, water molecules can exist in three distinct forms: within the cavities formed by the structure (zeolitic water); coordinated in Fe^III^ sites (coordination water); and linked to the coordinated water molecules through hydrogen bonding (lattice water).^16,49^ Using a heating rate of 3° min^–1^, the literature reports the loss of zeolitic water and lattice water at temperatures below 200 °C, leaving coordination water molecules in the compound structure. These remaining water molecules are probably released at a temperature above 200 °C.^50^ In the TGA curves obtained in the present work at a heating rate of 10° min^–1^ (Figure 6), this same behavior was observed, so the degrees of hydration of the synthesis products suggested formula in Table 4 must be higher.

Comparing the experimental and theoretical values (Table 4), of the second mass loss, related to cyanide ions, a lower percentage than expected was observed in Sigma-Aldrich Prussian blue and the synthesized products (except for A1 and I2). This variation suggests the occurrence of a greater quantity of structural defects in the crystal lattice of the compounds.^51^ The lower % mass of the second loss may be due mainly to two factors: the presence of impurities such as amorphous carbon and or aluminum hydroxide and a greater number of structural vacancies generated by the replacement of cyanide and Fe^II^ ions by water molecules that coordinate in Fe^III^ sites. These structural defects occur randomly in the crystal lattice, presenting a unit cell with a smaller number of atoms, and the molecular formula proposed for Prussian blue is an approximation.^38^

The third mass loss (690–900 °C) present in the TGA curve of sample I6 is possibly related to aluminum compounds, mainly Al(OH)_3_, corroborating the estimated value of aluminum (15.7%) by EDXRF. At a temperature lower than 350 °C, aluminum hydroxide transforms into the AlOOH species, which will later form Al_2_O_3_ as a residue at temperatures above 550 °C.^52^

**Figure 7:**
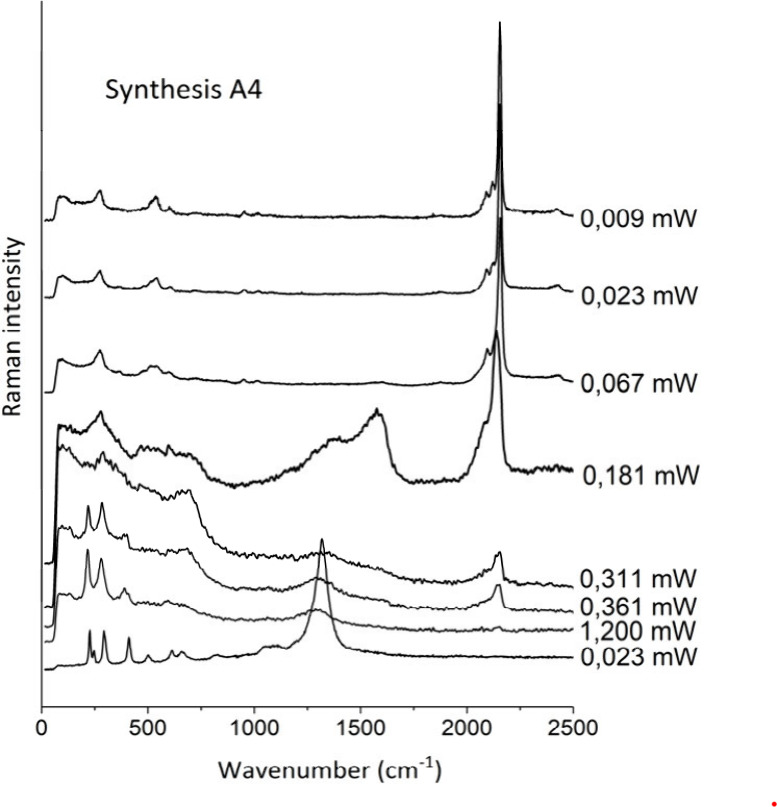
Laser-induced degradation spectra by Raman spectroscopy of sample A4.

Theoretically, the final residue of the Prussian blue thermal decomposition is hematite (eq 1), while in the soluble form of Prussian blue, containing potassium, the final residue of the thermal decomposition will possibly be a mixture of potassium and iron oxides (eq 2).^47,53^ For sample I6, containing Al(OH)_3_ impurity, the final residue might be the mixture of aluminum and iron oxides (eq 3).
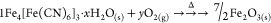
1
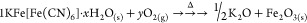
2
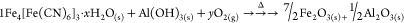
3The thermal decomposition (TGA) residues of all samples were analyzed by Raman spectroscopy. Hematite (Fe_2_O_3_) was identified as the final degradation product of Prussian blue (Figure S6), with characteristic bands at 226 cm^–1^, 246 cm^–1^, 295 cm^–1^, 411 cm^–1^, 501 cm^–1^, 613 cm^–1^, 660 cm^–1^, and 1318 cm^–1^.^54^ The same product was obtained by laser-induced degradation of all PB synthesized (Figure 7).

The low-frequency region in the Prussian blue Raman spectrum (100–1000 cm^–1^) presents vibrational bands related to the lattice structure of the compound. The characteristic bands of Prussian blue in this range fall at ∼275 and 360 cm^–1^, corresponding to the δ(Fe-CN-Fe) deformation modes, and 535 and 604 cm^–1^ (corresponding to the ν(Fe–C) modes of the lattice).^55,56^ Thus, changes in the bands that fall in this region correspond to degradation due to the loss of the crystalline structure of the compound, which began to occur at 0.067 mW of power.

The region 2070–2200 cm^–1^ provides information about the vibrations associated with iron and cyanide ions. In this range, bands are observed at ∼2155 cm^–1^ (corresponding to the A_1g_ mode of the ν(CN) stretching in [Fe^II^–CN-Fe^III^]) and 2090 cm^–1^ (corresponding to the E_g_ mode of ν(CN) in [Fe^II^–CN-Fe^III^]).^56,57^ Because it presents high sensitivity, the most intense Prussian blue band may undergo displacement induced by the incident radiation, and it is suggested to use a laser power range lower than 0.06 mW^56^.

Using Raman spectroscopy, the literature reports the presence of a band at ∼2123 cm^–1^, inferred to be ν(CN^–^) in the soluble form of Prussian blue.^56,58^ This band may be associated with the interaction between cyanide ions and cations of the compound structure. In the spectra obtained from the products synthesized in the present work, this band appeared with low intensity in samples A4 (Figure 7), A2, I6, and I10 (Figure S7). However, in most cases this band appears as a shoulder of the band at ∼2155 cm^–1^, being difficult to identify.

The presence of amorphous carbon (∼1350 cm^–1^ and ∼1600 cm^–1^) with Prussian blue was observed in the synthesized products even using low laser power (Figure S8), which may be due to the incomplete combustion of organic matter during the calcination step. Thus, amorphous carbon, alum, aluminum hydroxides, and KCl, identified in the analyses discussed above, are impurities that indicate a pigment obtained from an 18th-century methodology.

Although the stoichiometry involved in the ancient Prussian blue syntheses is not trivial, the formation and trapping of cyanide ions during the calcination step are crucial for a successful synthesis.^21,23,33,59,60^ During the calcination of organic matter, hydrocyanic acid is formed. Subsequently, these cyanide ions form potassium cyanide and, to a lesser extent, Fe(CN)_2_, as suggested in eqs 4 and 5.
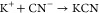
4
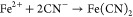
5
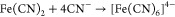
6
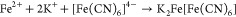
7
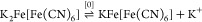
8
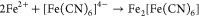
9
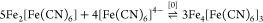
10For the formation of Fe(CN)_2_, iron sources other than animal matter are required, since dried ox blood and blood meal, a source for the formation of cyanide ions, presented low iron percentages, in the order of ∼0.4%. Other possible iron sources highlighted in several treatises from the 18th-century are iron vessels and earth pots for calcination. It is observed that the syntheses performed with an iron vessel presented higher yields compared to the syntheses performed in an alumina vessel (Table 1), corroborating the hypothesis of the formation of Fe(CN)_2_ during calcination and that a second iron source would help in the fixation of cyanide ions, as proposed in eq 4.

The calcination products (eqs 4 and 5) when dissolved in water, together with the Fe^2+^ ions from the iron(II) sulfate/alum solution, would react to give rise to hexacyanidoferrate(II) ions (eq 6). For the soluble form of Prussian blue, these ions would combine with the K^+^ ions and the Fe^2+^ ions to form the white Berlin White complex (eq 7). This complex would be rapidly oxidized by the oxygen in the air to produce the Prussian blue pigment (eq 8), which was visualized during the syntheses when the white precipitate immediately formed by the mixture of the calcined and iron(II) sulfate/alum solutions quickly changed to green. The proposed formation of Prussian blue in the insoluble form (eqs 9 and 10) follows the same reasoning as that for the soluble form.

The different proportions of reagents found in historical syntheses are probably associated with the difficulty in determining the reaction stoichiometry due to the fixation of cyanide ions in the calcination step. When the fixation of these ions does not occur efficiently, there will be an excess of Fe^2+^ ions in the solution. The yellowish color presented by the supernatant of the mixture between the calcination filtrate and the ferrous sulfate/alum solution in the syntheses carried out may be due to the formation of hydroxides and later oxides of oxidized iron. The formation of aluminum hydroxides is also expected in this step due to the use of alum. Thus, the amount of HCl used in the last step of the synthesis of Prussian blue is also decisive in explaining the identified impurities and the reaction yields. The use of iron vessels alone does not explain the low yields obtained and the discrepancy observed with the yields described in the 18th-century recipes, which leads one to believe that for a viable commercial production of this pigment in the 18th-century, there were many impurities associated with Prussian blue such as amorphous carbon and aluminum hydroxides.

## 4 Conclusion

A historical review of the methodologies used to manufacture Prussian blue until the beginning of the 19th century is presented in this work. They utilized various animal materials, different methods for obtaining alkali, and different reagent proportions evidencing the development of chemistry during the 18th century.

In this article, Prussian blue was synthesized based on the methodologies described by Dossie (v.1 and v.2, 1758). Among the literature consulted, this is the first work reported using materials indicated in the 18th century, such as ash alkali and iron vessels in the calcination stage. In the final product of the leaching of Candeia wood ash, various compounds such as K_2_CO_3_, KHCO_3_, K_2_SO_4_, KCl, K_2_O, and KOH were identified. Nonetheless, it was observed that the origin of the alkali and the animal matter did not significantly affect the products formed in Prussian blue syntheses. Regarding the time and maximum temperature of calcination, it was observed that these parameters depend on the material of the crucible used. However, a temperature of 400 °C and 90 min are necessary for Prussian blue formation.

The yields obtained were lower than those described in 18th-century literature, highlighting the complexities of the calcination step in the PB syntheses. The iron vessel used for calcination increases the yield, but this and the optimization time and temperature were insufficient to give the yields described by Brown (1724) and Dossie (1758) of approximately 20%. Only synthesis I6 (1:1–blood: alkali) produced quantities of pigment close to those found in historical sources but with the greatest impurities. The pigment is associated with aluminum and sulfur compounds.

The amount of HCl used is important to solubilize the Prussian blue synthesis byproducts, and in the 18th-century methodology, it is ambiguous. Determination of solution concentrations was only possible after the consolidation of atomic theory and the ideas of Dalton (Law of multiple proportions, 1807) and Proust (Law of definite proportions, 1797).

The Prussian blue produced for commercialization in the 18th century had likely the pigment adsorbed to aluminum hydroxide. The impurities identified in the Prussian blue, such as amorphous carbon, KCl, alum, sulfate/sulfide compounds, and aluminum hydroxide, can be used as evidence of a pigment obtained using 18th-century methods. These impurities could affect the pigment degradation process and color fading and help aid in dating cultural heritage.

Since Prussian blue is a radiation-sensitive pigment, laser powers below 0.067 mW should be used in Raman spectroscopy analysis.

## Supporting Information Available

Table of Prussian blue recipes in bibliographic sources, transcription of Dossie’s methodology (v.1 and v.2), quantities of reagents used in the synthesis, instrumental parameters, ash alkali and Prussian blue diffraction patterns, Prussian blue infrared and Raman spectra, and BSE and SE images by SEM (PDF)
