# Supplementary material for: Syntheses of Prussian Blue Pigment Following 18th-Century Methodologies: Factors Influencing Product Purity and Syntheses Yields
Source: ACS Omega. 2025 Mar 13;10(11):11375–85. doi: 10.1021/acsomega.4c11328 (PMC11947781; doi:10.1021/acsomega.4c11328)
Supplement: Supplementary file 1 — ao4c11328_si_001.pdf [file ao4c11328_si_001.pdf]

Supplementary Material: Syntheses of Prussian blue pigment following 18th-century methodologies: factors influencing product purity and syntheses yields

Authors: Mônica Grôppo Parma[a], Heloísa Beraldo[a], Isolda de Castro Mendes[a,*]

[a] Departamento de Química, Instituto de Ciência Exatas, Universidade Federal de Minas Gerais, Av. Antônio Carlos, 6627, 31270-901, Belo Horizonte, Minas Gerais, Brazil.

* Corresponding author

E-mail: monicaparma@ufmg.br, hberaldo@ufmg.br, isolda@ufmg.br*.

1. **Introduction**
2. **Materials and Methods**

2.1 Ash alkali

2.2 Prussian blue

Table S1. Prussian blue recipes in 18th and 19th-century bibliographic sources. Table adapted from Kirby and Saunders (2004).[1]

Reference	Initial matter	Alkali	Iron salt	Alum	Notes
Woodward, 1724[2]	4 dried blood	4 crude tartar<br>4 crude nitre	1 green vitriol calcined	8	HCl (2-3)
Brown, 1724[3]	4 dried blood	4 sal tartari	Varies	Varies	By the proportion given by Woodward, it yields 1 pound or a little more.
Shaw, 1734[4]	4 dried blood	4 crude tartar<br>4 crude nitre	1 green vitriol calcined	8	-
Crökern, 1736[5]	x dried blood	6 tartar<br>6 salpetre	1 English vitriol calcined	6	-
Dossie, 1758, v.1[6]	3 dried blood	1 pearl ashes	1 green vitriol	2	HCl (2)
Dossie, 1758, v.2[6]	8 dried blood	8 pearl ashes	1 copperas/vitriol calcined	16	HCl (3)<br>It yields 3 ounces for every pound of ash. It is said to be the original recipe published by Woodward.
Hellot, 1762[7]	3 dried blood	3 crude tartar<br>3 potash<br>1,5 salpetre	2 green vitriol	8	-
	3 dried blood	3 ashes (*cendres gravelées*)<br>2 crude tartar<br>1,5 salpetre	2 green vitriol	8	-
	3 dried blood	3 quick lime<br>2 crude tartar<br>1,5 salpetre	1,5 green vitriol	6	-
	3 dried blood	3 quick lime<br>2 crude tartar<br>1,5 calcined nitre	1 green vitriol	4	-
	3 dried blood	4,5 quick lime	1 green vitriol	4	-

		2 crude tartar<br>1,5 salpetre			
	3 dried blood	6 quick lime<br>2 crude tartar<br>1,5 calcined nitre	1 green vitriol	4	-
Guidotti, 1764[8]	1 dried blood	1 ashes salt	-	4+4	It doesn't use acid.
D'Apligny, 1779[9]	8 dried blood	8 potash	3 vitriol of Mars	4	-
Anonymous, 1785[10]	3 dried blood	1 pearl-ashes	1 green vitriol	2	Same procedure as ref. 6. It states that animal or vegetable matter can be used.
Seabra, 1788[11]	x sangue de boi dessecado	2 nitro fixado pelo tartaro ou 2 potassa pura	1 sulphurato de ferro	2 sulphurato argilloso	-
	45 cornos, unhas, couro, cabelos e outras matérias animais	20 potassa	6 sulphurato de ferro	40 sulphurato d'argilla	It doesn't use acid.
	x prussiato de potassa prepared by the first or second method		2 ounces sulphurato de ferro (first method)<br>30 parts sulphurato de ferro (second method)	-	Use muriatic acid or sulfuric acid or nitric acid.<br>Add the acid together with the sulphurato de ferro solution.
Secrets concernant les arts et métiers, 1791[12]	2 dried blood	2 crude tartar<br>2 crude nitre	1 vitriol of Mars	4	-
Hochheimer, 1792[13]	8 dried blood	8 potash	3 iron vitriol	4	-
	100 animal charcoal	100 potash	25 green vitriol<br>6-8 pound old iron	100	-
	15 calcined hoofs	20 potash	6 English vitriol	40	-
	36 hoofs	16 French tartar<br>25 potash	5 English vitriol	16	-
Hochheimer, 1794[14]	32-37 calcined hoofs	50 clean potash	12 green vitriol	80-100	-
Weber, 1793[15]	20 calcined hoofs	18 raw potash	5 ou 6 green vitriol	8 ou 10	-
	14 calcined hoofs	13 pure potash	5 green vitriol	15	-
Anonymous, 1794[16]	2 dried blood	2 crude tartar<br>2 nitre	1 vitriol of Mars	4	-
	4 dried blood	4 crude tartar<br>4 salpetre	1 English vitriol calcined	8	"In England they make a blue as beautiful as that of Prussia, using charcoal instead of ox blood, which creates a greater quantity of blue and is also darker."
Massoul, 1797[17]	2 dried blood	1 tartar alkali	1 Martial vitriol	3	-
Bachhoffner, 1837[18]	x horns and hoofs of animals, dried blood, or even	x pearlash (carbonate of potass)	1 sulphate of iron	2	-

	leather shavings				
Thompson, 1837[19]	2 coke, cinders or coal<br>1 iron turning	2 potash or pearlash	1 copperas	-	"… expose the whole for half an hour in an open fire to a full red heat, stirring the mass occasionally. During the process, little jets of purple flame will be observed to arise from the surface of the mixture; when these have almost ceased to appear, which will happen in about the time specified, the whole must be removed from the fire, and allowed to cool…"
	x coke or small coal<br>x iron turning	x nitrate of potash		-	"... in this case, the nitrogen is derived from the decomposition of nitric acid of the nitrate of potash, for the experiment succeeds equally well in a close vessel. In these experiments, soda may be substituted for potash without affecting the result…"

The Handmaid to the Arts Teaching – Robert Dossie (1758), v.1, p.78

" The Prussian blue may be prepared in perfection by the following process. "Take of blood any quantity; and evaporate it to perfect dryness. Of this dry blood, "powdered, take six pounds, and of the best pearl-ashes two pounds: mix them well "together in a glass or stone mortar; and then put the mixt matter into large crucibles or "earthen-pots; and calcine it in the furnace described, p. 22; the top of the crucible or "pot being covered with a tile, or other such convenient thing, but not luted. The "calcination should be continued, so long as any flame appears to issue from the matter; "or rather till it become very slender and blue; for if the fire be very strong, a small "flame would arise for a very long time. When the matter has been sufficiently calcined, "take the vessels which contain it out of the fire; and, as quickly as possible, throw it "into two or three gallons of water; and, as it soaks there, breack it with a wooden "spatula, that no lumps may remain. Put it then in a proper tin-vessel, and boil it for the "space of three quarters of an hour or more; and filter it while hot through paper in the "tin cullenders described, p. 27; and pass some water through the filter when it is run "dry, to wash out the remainder of the lixivium of the blood and pearl-ashes; the earth "remaining in the filter may be then thrown away. In the mean time, dissolve of clean "alum four pounds, and of green vitriol or copperas two pounds, in three gallons of "water. Add this solution gradually to the filtered lixivium, so long as any affervescence "appear to arise on the mixture; but when no ebullition or fermente follows the "admixture, cease to put in more. Let the mixture then stand at rest, and a

green powder "will be precipitated: from which, when it has thoroughly subsided, the clear part of the "fluid must be poured off, and fresh water put in its place, and stirred well about with "the green powder; and, after a proper time of settling, poured off like the first. Take "then of spirit of salt double the weight of the green vitriol which was contained in the "quantity of solution of vitriol and alum added to the lixivium, which will soon turn the "green matter to a blue colour; and, after some time, add a proper quantity of water, "and wash the colour in the same manner, as has been directed for lake, ξc.; and when "properly washed, proceed in the same manner to dry it in lumps of covenient size."

The Handmaid to the Arts Teaching – Robert Dossie (1758), v.2, p.424

"The original recipe for the making Prussian blue, as published by Dr. Woodward.

"Take any quantity of blood, and evaporate it do dryness, continuing the heat till it become "black, but avoiding the burning any part of it to ashes. Powder the dry matter, and mix "it thoroughly with an equal weight of pearl-ashes, and calcine the mixture in an iron pot "or crucible, on which a cover is put. The calcination must be continued so long as the "matter emits any flame, the fire being raised to a considerable degree of heat at the end "of the operation, and the matter must be then powdered, and put, while yet hot, into "twelve times its weight of water, which must be again set on fire to boil for the space of "three quarters of an hour, or more. The fluid must then be filtered off through a thin "flannel bag, from the part remaining undissolved; through which remaining part fresh "water should be passed, before it be taken out of the filtering bag, to extract as much as "possible of the solution; and the water, thus passed through, should be added to the "quantity before filtered; after which, what is retained in the bag bay be thrown away. In "the meantime a solution should be made of allum and copperas calcined to whiteness, in "the proportion of two pounds of the allum, and two ounces of the calcined vitriol, to each "pound of the pearl-ashes used with the blood, which solution must be made by boiling "the allum and copperas in five times their weight of water, and then filtering them "through flannel or paper, where great nicety is required. When the solution of the allum "and copperas is thus prepared, it must be added to the lixivium filtered off from the "calcined blood and pearl-ashes, from which mixture, the precipitation of a blackish "green matter will soon ensue. After the precipitated matter has subsided to the botton of "the vessel, and the fluid appears clear over it, separate it from the green sediment, first "by pouring off all that will run clear out of the vessel, and afterwards by straining off the "remainder, and the put the green matter again into a vessel that will contain as much "fluid as it was before mixt with. Add spirit of salt to it afterwards, in the proportion of "six ounces to every pound of the pearl-ashes used, and the green matter will then soon "appear to be converted into a beautiful blue. Water must then be added to wash off the "spirit of salt, which must be renewed several times till it come off perfectly sweet, and "the last quantity must then be strained off, and the blue sediment dried in lumps of a "moderate size. The produce will be about three ounces for every pound of the pearl-ashes "employed."

This recipe was omitted in the first part of this work for want of room, and another inserted, where the proportions of the ingredients are more accurately adapted to each other, in order to make a saving in the expence. But this recipe will produce an equally fine colour, and if the produce be desired to be made either of a lighter or

darker hue, it may be done by increasing the proportion of the pearl-ashes to the blood to give a lighter kind, or the spirit of salt to the pearl-ashes to give a deeper kind; but the quantity will in the latter case be proportionably diminished.

The straining or filtering the lixivium through flannel is not so good a method as the doing it through paper, especially where the colour is wanted of a very great brightness and purity, and the water is best separated from the great sediment first produced, and afterwards from the blue one by the same means; but in these cases a fine linen cloth much worn, though whole, should be laid over the paper. The colour, when reduced to a proper consistence, may be laid on chalk-stones to dry, and a moderate heat may be also used for greater expedition, when required; but great care should be taken not to burn the matter. The calcination may be performed in a reverberatory furnace, such as is used by the chymists, or in the furnace where metals are melted; for the crucible or pot containing the matter may either be surrounded by the coals, or placed over them, provided a sufficient heat be given to it. But where larger quantities are to be calcined, they may be very cheaply and commodiously managed in the potters or tobacco-pipe-makers furnaces, being put into them along with the earthen ware and pipes. And if the calcined matter in such case cannot be conveniently necessary, if the matter be well powdered afterwards, before it be put into the water."

Table S2. Quantities of reagents used in the syntheses.

Reference	Dried ox blood (g)	Alkali (g)	Green vitriol (g)	Alum (g)	Spirit of salt* (g)
Dossie, 1758, v.1	30	10	10	20	20
Dossie, 1758, v.2	30	30	3.75	60	11.25

*HCl 37% solution mass.

Eighteenth-century chemists knew how to purify solid substances, but determining the concentration of solutions was only possible after the consolidation of atomic theory and the ideas of Dalton (Law of multiple proportions, 1807) and Proust (Law of definite proportions, 1797), which form the basis of stoichiometry. For this reason, in the 18th-century recipes researched, we found the quantity of hydrochloric acid described in mass and without reference to concentration[20]. Thus, to reproduce the syntheses of Prussian blue, we found it more appropriate to use the mass of the HCl solution.

The alum + iron sulfate solution was prepared by weighing the salts in a beaker and adding 150 mL of deionized water for Dossie v.1 synthesis, and 400 mL for Dossie v.2 synthesis. The mixtures were left under constant stirring for one hour, filtered, and added to the calcination filtrate.

2.3 Raman light scattering spectroscopy

Identification and degradation analyses were conducted using a Confocal Raman Microscope (WITec), model Alpha300RA. Instrument calibration was verified before each measurement using a silicon reference standard (520 cm^{-1}). The measurements were performed with a 100x objective lens, a 532 nm wavelength laser, a 600 g/mm diffraction grating, and a spectral band centered at 2100 cm^{-1}, achieving a spectral resolution of 3 cm^{-1}.

Raman spectra for studying the degradation process were acquired by incrementally varying the laser power with the following values: 0.009 mW, 0.023 mW, 0.067 mW, 0.181 mW, 0.311 mW, 0.361 mW, 1.200 mW, and 2.385 mW. These measurements were carried out under 10 scans with an integration time of 20 seconds per scan. Data processing was performed using Project FOUR 4.1 software. Spikes caused by cosmic rays removed from the spectra using the "Cosmic Ray Removal" (CRR) function.

2.4 Fourier transform infrared spectroscopy (FTIR)

The FTIR analyses were conducted using a Perkin Elmer FTIR RX I instrument, with a spectral range of 4000 to 400 cm^{-1}. Spectra of Prussian blue samples were obtained in transmittance mode. The analyses were performed with 13 mm KBr pellets, utilizing 32 scans and a resolution of 4 cm^{-1}. For alkali ash samples, the attenuated total reflection (ATR) mode was employed, using a diamond crystal, with 64 scans and a resolution of 4 cm^{-1}.

2.5 Energy-dispersive X-ray fluorescence (EDXRF)

Semiquantitative elemental analyses of Prussian blue and alkali ash samples were conducted using a DELTA XRF (Olympus) portable instrument equipped with a Rhodium anode and an SDD detector. The fundamental parameter method was employed for elemental quantification. Elements present at concentrations below 0.1% were classified as trace elements. Measurements were performed in Mining Plus mode without vacuum, under two conditions: 30 s at 100 μA and 40 kV, and 60 s at 200 μA and 10 kV.

2.6 Scanning electron microscopy/energy-dispersive x-ray spectrometry (SEM-EDS)

Backscattered electron (BSE) and secondary electron (SE) images, as well as X-ray fluorescence data (EDS), of Prussian blue and alkali ash samples were acquired using a HITACHI TM4000Plus Scanning Electron Microscope, coupled with an Oxford Instruments EDS system. Powder samples were mounted on carbon tape and analyzed under low vacuum conditions.

2.7 X-ray diffraction (XRD)

X-ray powder diffraction of Prussian blue and alkali ash samples was performed using an Empyrean II diffractometer with Cu-Kα radiation (1.541874 Å). The instrument was operated at 45 kV and 40 mA. Scanning was conducted over a 2θ range of 4° to 80°, with a step size of 0.05°. The resulting diffractograms were analyzed using the software "Match!" version 3.15 and compared with data deposited in the Crystallography Open Database (COD).

2.8 Thermogravimetric analysis (TGA)

Thermogravimetric analysis of Prussian blue samples was conducted using a Shimadzu 60H instrument, with an alumina crucible as the sample holder. The measurements were performed under an air atmosphere with a flow of 50 mL min^{-1} and a heating rate of 10 °C min^{-1} from room temperature to 900 °C.

3 Results e discussion

3.1 Ash alkali

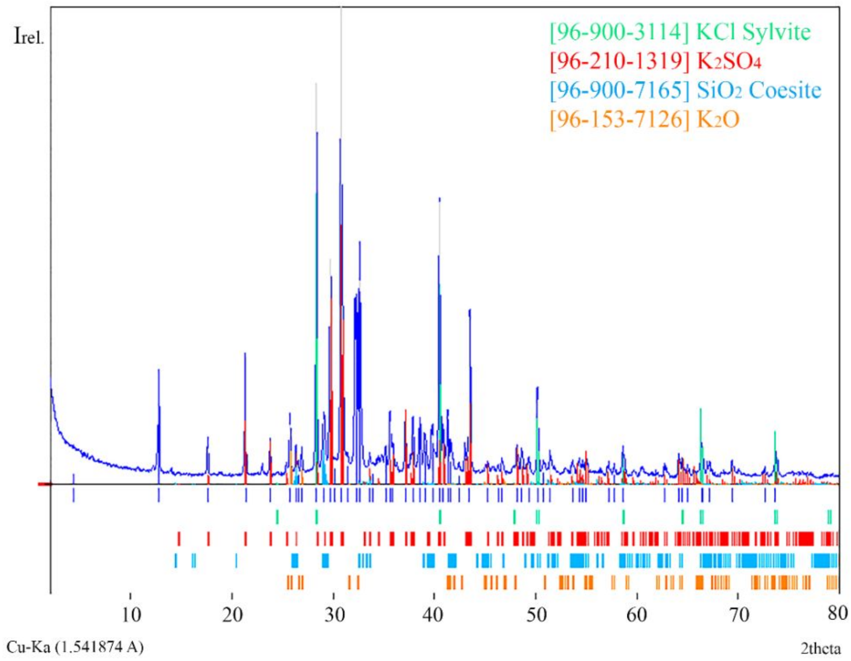

Figure S1. Ash alkali diffraction pattern.

3.2 Prussian blue

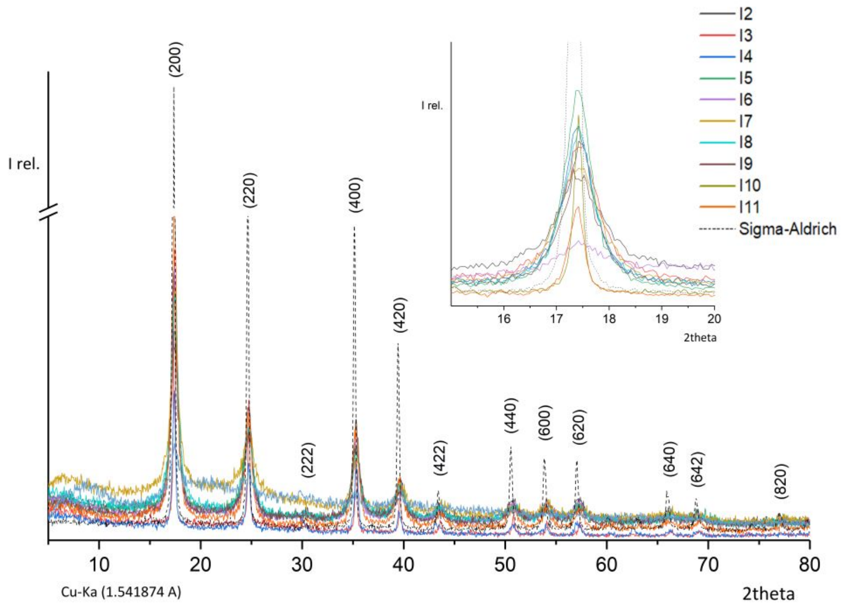

Figure S2. Diffraction patterns of synthesized samples in iron containers and Sigma-Aldrich Prussian blue.

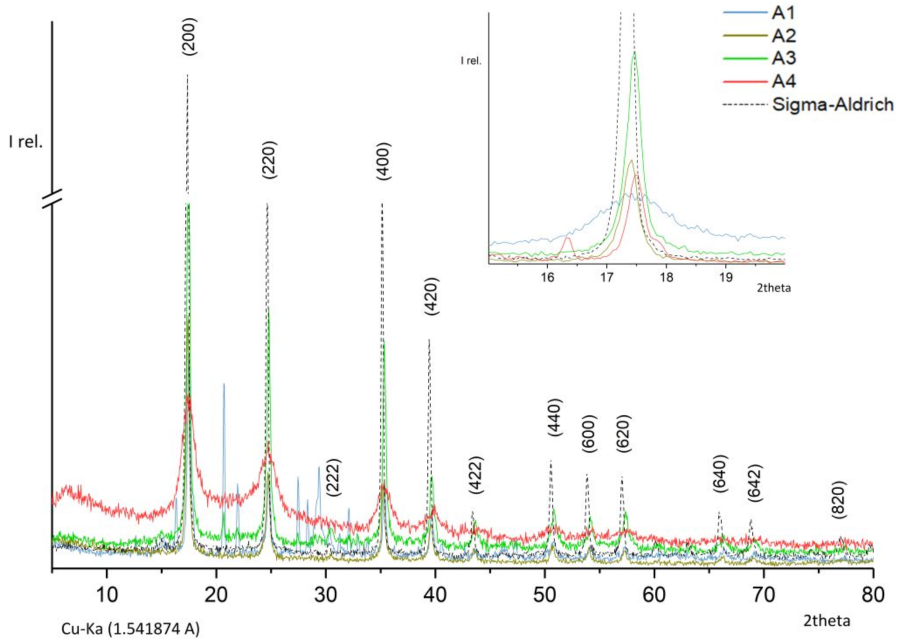

Figure S3. Diffraction patterns of synthesized samples in alumina containers and Sigma-Aldrich Prussian blue.

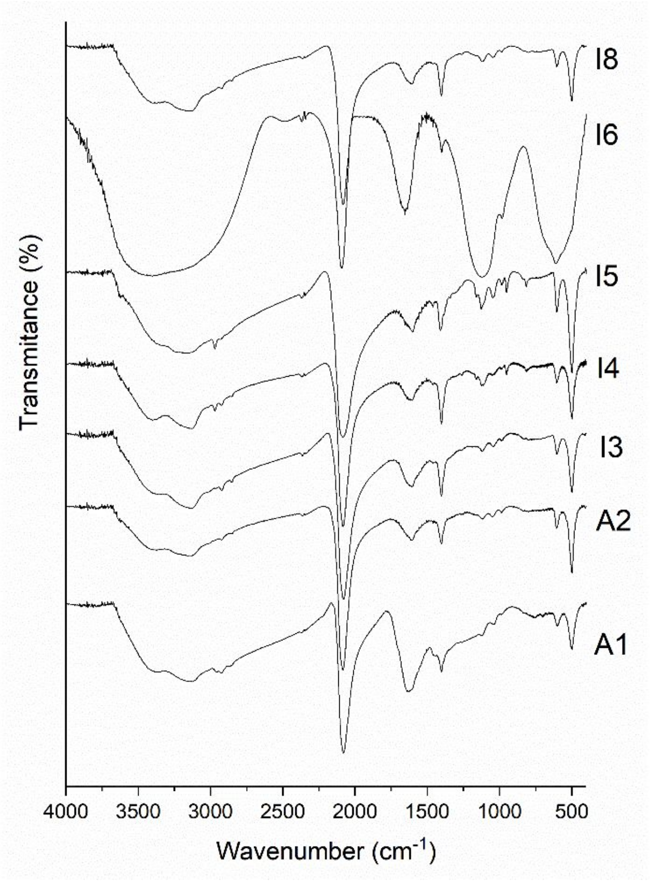

Figure S4. Baseline-corrected infrared spectra (KBr pellets) of samples A1, A2, I3, I4, I5, I6 and I8.

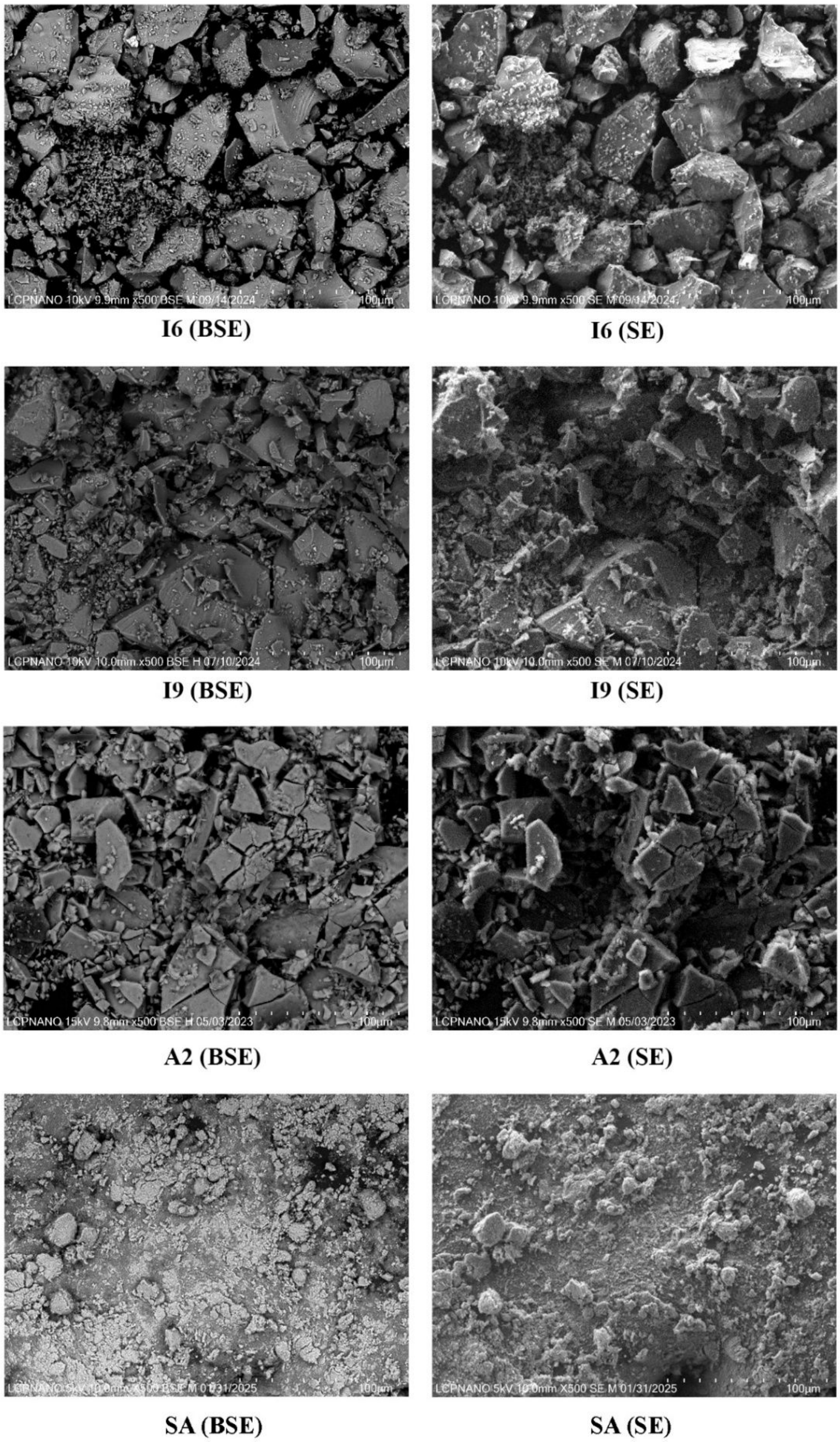

Figure S5. BSE and SE images at 500x magnification, obtained by SEM, of samples I6, I9, A2, and SA (Sigma-Aldrich).

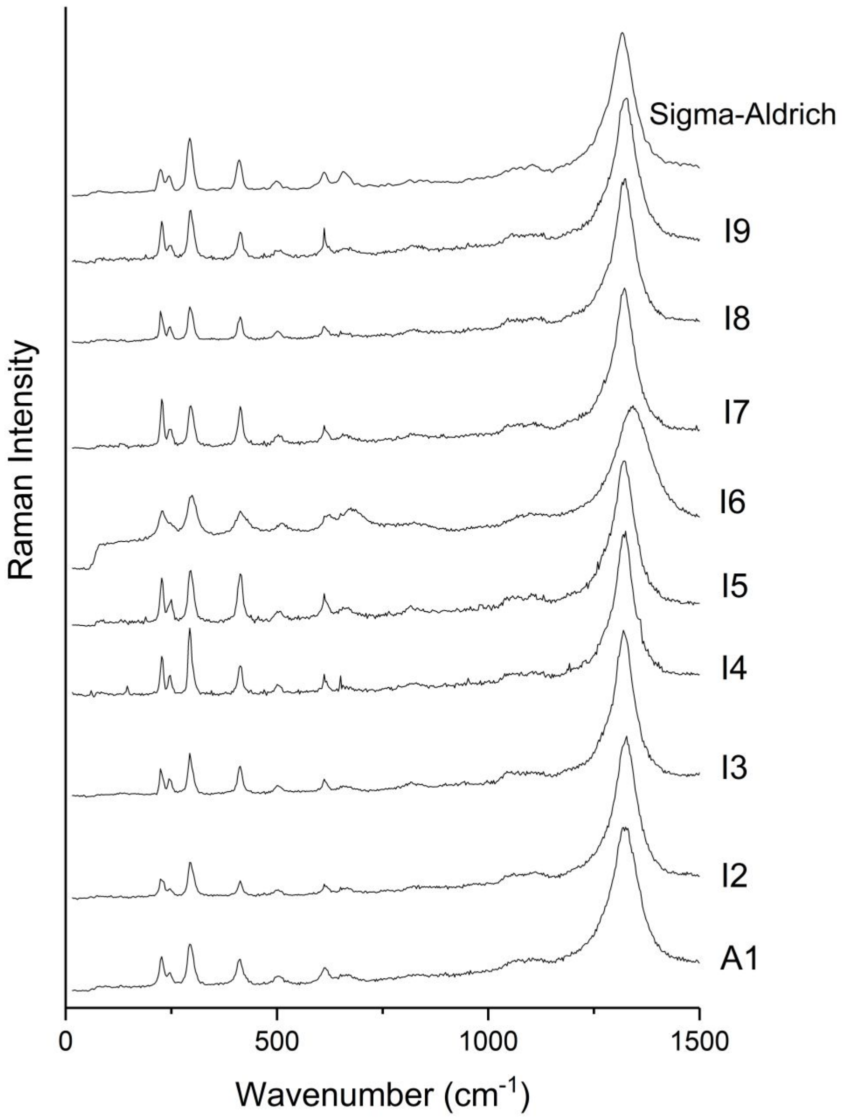

Figure S6. Raman spectra of the residues by TGA. Spectra were acquired with a 532 nm laser and a power of 0.023 mW.

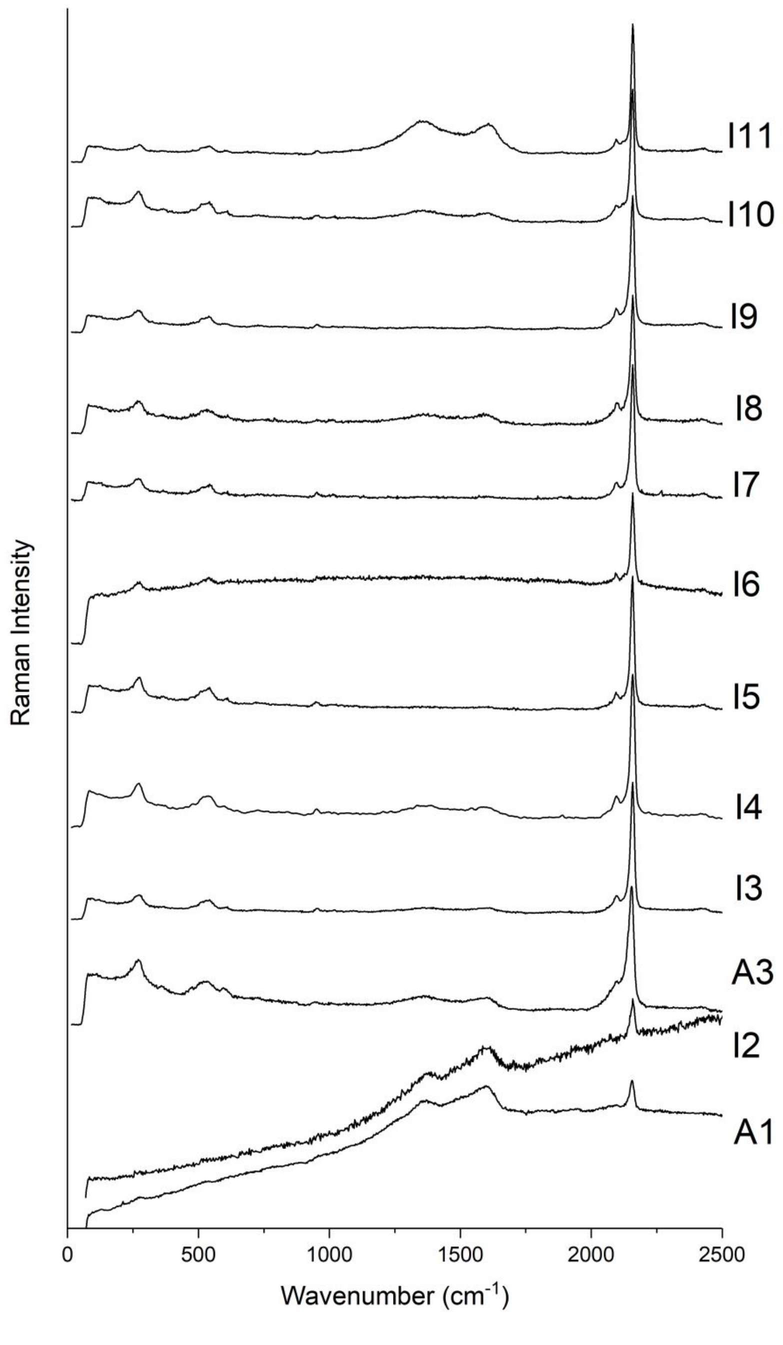

Figure S7. Raman spectra of synthesized Prussian blue. Spectra were acquired with a 532 nm laser and a power of 0.023 mW.

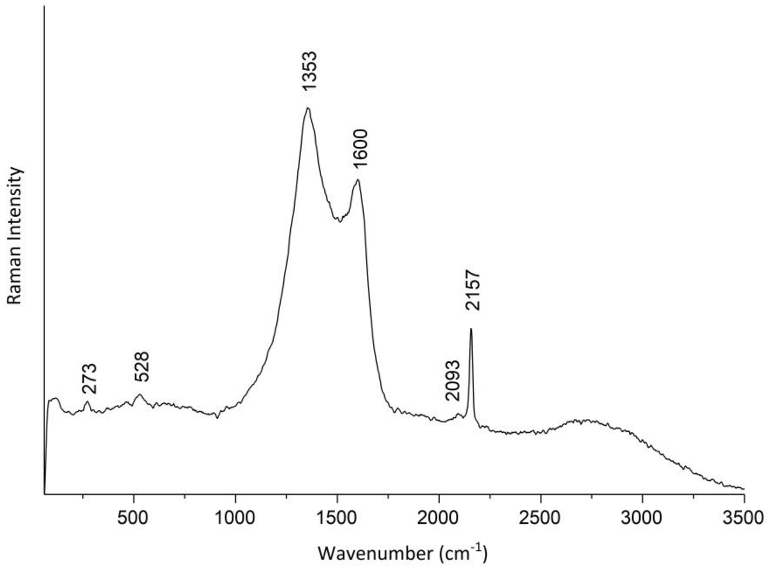

Figure S8. Raman spectrum of sample A2. Spectrum was acquired with a 532 nm laser and a power of 0.023 mW.

Acknowledgments

The authors gratefully acknowledge CAPES, CNPq, and FAPEMIG (RGP Grant) for student grants and financial support, and PPG-Química, GruTam (Departamento de Química, Universidade Federal de Minas Gerais), and LCPNano and LabCri (Departmento de Física Universidade Federal de Minas Gerais).

References

(1) Kirby, J.; Saunders, D. Fading and Colour Change of Prussian Blue: Methods of Manufacture and the Influence of Extenders. *Natl. Gall. Tech. Bull.* **2004**, *25*, 73–99.

(2) Woodward, J. Præparatio Cærulei Prussiaci Ex Germaniâ Missa Ad Johannem Woodward, M. D. Prof. Med. Gresh. R. S. S. *Phil Trans R Soc London* **1724**, *33* (381), 15–17.

(3) Brown, J. V. Observation and Experiments upon the Foregoing Preparation. *Philos. Trans. R. Soc. London* **1724**, *33* (381), 17–24. https://doi.org/10.1098/rstl.1724.0006.

(4) Shaw, P. *Chemical Lectures, Publickly Read at London, in the Years 1731 and 1732; and since at Scarborough, in 1733; for the Improvement of Arts, Trades, and Natural Philosophy*; J. Shuckburgh: London, 1734.

(5) Crökern, J. M. *Der Wohl Anführende Mahler*; U. Schiessl: Jena, 1736.

(6) Dossie, R. *The Handmaid to the Arts Teaching*; J. Nourse: London, 1758.

(7) Hellot, M. Sur La Préparation Du Bleu de Prusse. In *Histoirede l'Académie Royale des Scienes. Année MDCCLVI*; L'imprimerie Royale: Paris, 1762; pp 53–59.

(8) Guidotti, A. M. A. *Nuevo Trattato Di Qualsivoglia Sorte Di Vernici*; Lelio della Volpe: Bologna, 1764.

(9) D'Apligny, M. L. P. *Traité Des Couleurs Matérielles*; Saugrain & Lamy, Et Barrois: Paris, 1779.

(10) Anonymous. *The Artist's Assistant, in the Study and Practice of Mechanical Sciences*; Printed for the author and sold by G. Robinson and M. Swinney Birmingham: London, 1785.

(11) Seabra, V. C. de. *Elementos de Chimica*; Na real officina da Universide: Coimbra, 1788.

(12) *Secrets Concernant Les Arts et Métiers*, Tomo 2.; Chez Bossange & Compagnie, Libraires & Commissionnaires: Paris, 1791.

(13) Hochheimer, C. F. A. *Chemische Farben-Lehre, Oder Ausführliche Unterricht von Bereitung Der Farben Zu Allen Arten Der Malerey*; Graffschen Buchhanblung: Leipzig, 1792.

(14) Hochheimer, C. F. A. Chemische Farben-Lehre, Oder Ausführliche Unterricht von Bereitung Der Farben Zu Allen Arten Der Malerey; Graffschen Buchhanblung: Leipzig, 1794.

(15) Weber, J. A. *Chemische Erfahrungen Bey Meinem Und Andderen Fabriken in Deutschland*; Johann Ludwig Bebra: Neuwied, 1793.

(16) Anonymous. *Segredos Necessarios Para Os Officios, Artes, e Manufacturas, e Para Muitos Objetos Sobre a Economia Domestica*, Tomo 2.; Offic. de Simão Thadeo Ferreira, Ed.; Lisboa, 1794.

(17) Massoul, M. C. de. *A Treatise on the Art of Painting and the Composition of Colour*; T. Baylis: London, 1797.

(18) Bachhoffner, G. H. *Chemistry as Applied to the Fine Arts*; J. Carpenter and Co. Old Bond Street: London, 1837.

(19) Thompson, L. N° I . Preparation of Prussian Blue. *Trans. Soc. Instituted London, Encourag. Arts, Manuf. Commer.* **1837**, *52* (I), 24–27.

(20) Glauber, J. R. *The Works of the Highly Experienced and Famous Chymist, John Rudolph*

Glauber; Publick Good: London, 1689.
